# Integrated prevalence mapping of schistosomiasis, soil-transmitted helminthiasis and malaria in lakeside and island communities in Lake Victoria, Uganda

**DOI:** 10.1186/1756-3305-4-232

**Published:** 2011-12-13

**Authors:** Narcis B Kabatereine, Claire J Standley, Jose C Sousa-Figueiredo, Fiona M Fleming, J Russell Stothard, Ambrose Talisuna, Alan Fenwick

**Affiliations:** 1 Vector Control Division, Ministry of Health, Kampala, Uganda; 2 Wolfson Wellcome Biomedical Laboratory, Department of Zoology, Natural History Museum, London, SW7 5BD, UK; 3 Ecology and Evolutionary Biology, Princeton University, Princeton, NJ 08544, USA; 4 Schistosomiasis Control Initiative, School of Public Health, St. Mary's Campus, Norfolk Place, London W2 1PG, UK; 5 Disease Control Strategy Group/Molecular and Biochemical Parasitology Group, Liverpool School of Tropical Medicine, Pembroke Place, Liverpool, L3 5QA, UK; 6 University of Oxford/KEMRI/Wellcome Trust Research Programme, P.O. Box 43640, 00100, Nairobi, Kenya

**Keywords:** Neglected tropical diseases, *Schistosoma mansoni*, *Plasmodium falciparum*, hookworm, *Ascaris lumbricoides*, *Trichuris trichiura*, integrated control, mass drug administration

## Abstract

**Background:**

It is widely advocated that integrated strategies for the control of neglected tropical diseases (NTDs) are cost-effective in comparison to vertical disease-specific programmes. A prerequisite for implementation of control interventions is the availability of baseline data of prevalence, including the population at risk and disease overlap. Despite extensive literature on the distribution of schistosomiasis on the mainland in Uganda, there has been a knowledge gap for the prevalence of co-infections with malaria, particularly for island communities in Lake Victoria. In this study, nine lakeshore and island districts were surveyed for the prevalence of NTDs and malaria, as well as educational and health infrastructure.

**Results:**

A total of 203 communities were surveyed, including over 5000 school-age children. Varying levels of existing health infrastructure were observed between districts, with only Jinja District regularly treating people for NTDs. Community medicine distributors (CMD) were identified and trained in drug delivery to strengthen capacity. Prevalence levels of intestinal schistosomiasis and soil-transmitted helminthiasis were assessed via Kato-Katz thick smears of stool and malaria prevalence determined by microscopy of fingerprick blood samples. Prevalence levels were 40.8%, 26.04% and 46.4%, respectively, while the prevalence of co-infection by *Schistosoma mansoni *and *Plasmodium *spp. was 23.5%. Socio-economic status was strongly associated as a risk factor for positive infection status with one or more of these diseases.

**Conclusions:**

These results emphasise the challenges of providing wide-scale coverage of health infrastructure and drug distribution in remote lakeshore communities. The data further indicate that co-infections with malaria and NTDs are common, implying that integrated interventions for NTDs and malaria are likely to maximize cost-effectiveness and sustainability of disease control efforts.

## Background

There has been a recent renewed global interest to control neglected tropical diseases (NTDs), which include intestinal schistosomiasis and soil-transmitted helminthiasis (STH) [1,2]. This focus is timely given the huge burdens these parasites place on many populations, particularly in Africa. Moreover, the distribution of intestinal helminthes often overlaps with that of *Plasmodium falciparum*, the pathogenic agent causing the bulk of the world's fatal malaria cases [3]. While malaria control has received significant investments in the last decade which has resulted in significant disease burden reductions [4], the burden of NTDs in most African countries is little known. In Uganda, schistosomiasis mainly occurs around the large lakes and rivers, including Lake Victoria, affecting approximately 4 million people, while 16.7 million are estimated to be at risk from the infection [5]. Malaria remains the most devastating health problem in the country, causing an estimated 40,000 deaths each year, but our understanding of the real disease burden is still limited due to lack of reliable data [6].

Since 2000, an increasing number of countries have embarked on national control programmes against one or more of the NTDs and/or malaria, but rarely have these efforts been integrated to examine the overall burden of combined parasitic infections. We believe that on-going control initiatives should integrate multiple diseases, and that mapping and monitoring processes should be integrated to maximize the effectiveness of the limited resources dedicated to disease management.

The Global Network for Neglected Tropical Diseases (GNNTD: http://globalnetwork.org) has been instrumental in raising awareness and promoting advocacy relating to the NTDs. The initiative has also provided funding to national health programmes, through partner collaborations, in order to examine existing control components and broaden them as necessary, particularly to integrate treatment for more than one NTD. In Uganda, a programme for the control of intestinal schistosomiasis has been in place since 2003, coordinated by the Vector Control Division (VCD) of the Uganda Ministry of Health, with support from the Schistosomiasis Control Initiative (SCI; http://www.sci-ntds.org). Albendazole, for the treatment of STH, was included in this mass drug administration (MDA). The National Control Programme also included basic health education training and information on how behavioural change can reduce transmission, but no funding was available for improvement of sanitation facilities, water pumps or other health-related infrastructure.

SCI's involvement with the programme shifted focus in 2007 [7], when funding was taken over by USAID as part of a scaled-up, fully integrated approach to NTD control. The integrated NTD approach resulted in the inclusion of ivermectin and azithromycin as part of the preventive chemotherapy strategy [8,9]. However, malaria has not been integrated into these control efforts; the distribution of artemisinin-based combined therapies (ACTs) and insecticide treated bednets is coordinated by the Ministry of Health's Malaria Control Programme (MCP; http://www.health.go.ug/mcp/index2.html) with minimal involvement of the vector control division that is responsible for NTDs. As such, there is a lack of coordinated information regarding the co-prevalence of intestinal helminthes and malaria, yet such data are crucial to developing integrated control efforts that take into consideration the local context and risk factors.

In Uganda, past surveying and mapping efforts have identified a number of hotspots for intestinal schistosomiasis and STH; the shoreline of Lake Victoria is particularly at risk in view of the low levels of sanitation, high population density and extremely permissible water environments [10-12]. The number of islands in the lake, many relatively remote from the mainland, provide even greater challenges to control efforts, and require regular monitoring to ensure that control initiatives, including training of health personnel, drug delivery and MDA, reach the required targets. Here, we present the results of a first-ever wide coverage integrated schistosomiasis, STH and malaria survey among primary school-age children across nine districts bordering Lake Victoria, including island communities. The aim of the survey was to examine risk factors associated with these three diseases, including identifying existing health infrastructure, evaluating the prevalence of intestinal schistosomiasis, STH and malaria as well as ascertaining the prevalence of co-infections on the islands and in a sample of villages on the shoreline. Finally, we wanted to provide the best-evidence for developing and improving integrated national control strategies for under-served and less accessible communities.

## Methods

### Mapping of island communities and treatment infrastructure

**Figure 1:**
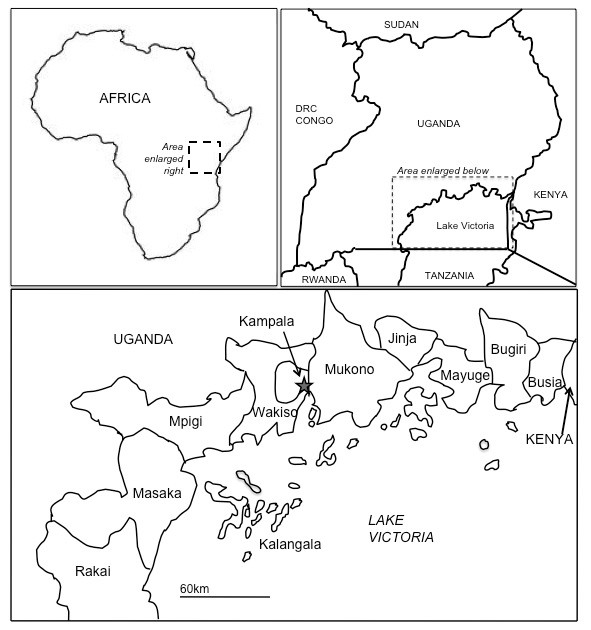
**Maps**. Maps showing the location of Lake Victoria and the districts surveyed in this study.

Between September 2009 and April 2010, Vector Control Division (VCD) teams comprising of scientists and technicians were joined by relevant district staff and visited a total of 203 villages scattered over 126 of the 150 inhabited islands in Lake Victoria, Uganda. At each location, the team identified if the island was inhabited and if so, the island's global-positioning system (GPS) coordinates were recorded with a hand-held GPS device (GPS III, Garmin, Olathe, USA). The visited islands are part of six administrative districts that surround Lake Victoria, namely Mpigi, Wakiso, Mukono, Jinja, Mayuge and Bugiri. The other districts surveyed included Kalangala, which consists solely of an island archipelago, and Rakai and Masaka, which have no islands but border the Lake (Figure 1).

At each location, the population estimates of the village and of the island were evaluated in order to determine numbers at risk of infection, as well as the amount of drugs required in each locality. The team additionally collected information on existing health infrastructure and availability of personnel who could be utilized during mass treatment campaigns.

### Training and capacity building

Since the island populations are often remote and difficult to reach, communities were first mobilized and sensitized to elect community volunteers, known as community medicine distributors (CMDs). These CMDs were trained about essential elements of disease transmission for schistosomiasis and STH, health benefits of treatment and the methods for control of these diseases. They were also practically trained on how to register the target population, deliver health education messages and determine and deliver praziquantel (PZQ) doses using a tablet pole. Finally, the CMDs were given guidance on when and where to refer cases for further management of side-effects during mass treatment campaigns. At least two CMDs per village were trained. The training involved CMDs from neighbouring villages so that by the end of the survey, every village on the islands had CMDs who delivered treatments during the survey and continued to treat even after the end of survey to achieve wide treatment coverage.

### Parasitological surveys

At each site, surveys were undertaken to assess prevalence of schistosomiasis (caused by *Schistosoma mansoni*), STHs (*Ascaris lumbricoides, Trichuris trichiura *and hookworm species) and malaria (*Plasmodium *spp.). In order to survey as many villages as possible, the Lot Quality Assurance Sampling protocol, which classifies the prevalence of infection using a small sample size [13], was followed. Thus a total of 15 children, with a roughly even mix of boys and girls, ranging in age from 10-14 years, were randomly selected in each village. If in the first village 8 boys and 7 girls were nominated, in the next village the numbers would be interchanged so that the final sample had an equal number of boys and girls.

Participants were asked to provide a stool, urine and fingerprick blood sample for parasitological diagnosis. Stool samples were used to create double Kato-Katz thick smears [14], which were read in the field by certified field technicians for counts of helminth eggs (*S. mansoni *and STH). Numbers of eggs per slide were recorded and multiplied by 24 to obtain a measure of the number of *S. mansoni *eggs per gram of faeces (EPG). Urine samples were used to conduct circulating cathodic antigen (CCA) urine lateral flow tests as an additional diagnostic tool to detect *S. mansoni *infections; these results are not shown here, but are published in part elsewhere, for example for Kalangala District [15]. The fingerprick blood was used to create double thin and thick blood smears on a glass slide, which were stained and read by other experienced technicians for presence and abundance of *Plasmodium *spp.

For mapping of intestinal schistosomiasis and malaria distributions, prevalence data from each geo-referenced village were entered into ArcGIS (ESRI, Redlands, USA). Each data point was coloured according to prevalence at that location. Co-infections were mapped using ArcGIS, and the prevalence pie charts drawn in PowerPoint 2010 (Microsoft Office 2010, Microsoft, Richmond, USA). The prevalence of intestinal schistosomiasis and malaria were mapped separately for all of the geo-referenced villages. Prevalence boundaries were determined by cut-off values for different treatment regimes, as per WHO recommendations [16].

### Demographical questionnaire and statistical analyses

Each child that agreed to participate in the parasitological survey was also asked to participate in a questionnaire, consisting of a series of short questions designed to gather data on demographical and socioeconomic factors that might relate to infection risk. The questions covered basic personal information, health information, residence information, household wealth questions, bednet use data, history of de-worming and information on absenteeism and recent illnesses (see Additional File 1 for a copy of the questionnaire used).

Since children were from different villages, the possibility of intra-correlation in the data cannot be discarded. Therefore, regression analyses were carried out taking into account within-village intra-correlation in the data using a generalized linear mixed model with multivariate normal random-effects (the random-effects of village in our case), with penalized quasi-likelihood (function glmmPQL in R) [17,18]. Models were established defining infected children as cases, i.e. infection status was treated as a binary variable (infected and not infected) and incorporating all variables from the questionnaire. The main model results presented in this article result from forwards and backwards stepwise regression to select the most parsimonious, yet adequate model using Akaike information criterion (AIC) values [19]. In addition, to test more directly for the effect of specific variables, such as absenteeism and bed net usage, new multivariate logistical regression models were constructed, also including village-level random-effects. For both stepwise and non-stepwise methods, all final models for infections in children controlled for gender and age. One exception to this methodology was the univariate model for socio-economic status (SES), which controlled for village-level random effects, but not sex or age. The variable SES was constructed by attributing each child a value of zero to five, depending on the number of variables present in the respective household: electricity, solar power, latrine, landline and mobile phone. SES was considered a continuous variable in the univariate model. For each variable, odds ratio (OR) and *P*-values were calculated, and a *P*-value < 0.05 was considered indicative of statistical significance.

### Ethical considerations

Prior to taking part in the survey, each child's parent or guardian was asked to provide informed written permission; in addition, each child was asked for informed oral consent and reminded that they could withdraw from the survey at any time. All participating children were offered treated with praziquantel (PZQ) for infection with *S. mansoni *and albendazole (ALB) for infection with STH. Children who tested positive for malaria were given appropriate artemisinin-based combined therapy (ACT). All treatment was dosed and administered by a community or a Vector Control Division nurse. Ethical approval for the study was granted by the NHS-LREC (Application 03.36) and the Uganda National Council for Science and Technology.

## Results

### Mapping of islands, treatment infrastructure and capacity building

Lake Victoria is comprised of 212 islands, of which 150 (70.8%) are inhabited by an estimated 220,736 people (Table 1). Most of the islands are situated in Mukono and Kalangala districts, which each have 84 islands. Wakiso district has 8 islands, Bugiri has 9 islands and Mayuge has 7 islands. Of those districts with islands, Jinja and Mpigi have the least number, at 3 and 4 islands respectively. All inhabited islands, save 23 in Kalangala and one in Mukono district, were surveyed. Thus a total of 126/150, (84.0%) islands were surveyed and altogether 203 communities on these islands were involved in the survey (Table 1).

**Table 1:** Numbers of islands surveyed and estimated total population of Lake Victoria islands

District	Total population	Number of islands	Number of islands inhabited	Number of islands surveyed	Number of villages surveyed
Bugiri	20,530	22	9	9	9

Jinja	14,22	3	3	3	3

Kalangala	44,200	84	60	37	61

Masaka	15,078	-	-	-	15

Mayuge	15,214	7	6	6	6

Mpigi	21,108	4	4	4	5

Mukono	52,963	84	60	59	74

Rakai	8,310	-	-	-	11

Wakiso	15,050	08	08	08	19

**Total**	**220,736**	**212**	**150**	**126**	**203**

Treatment infrastructure and MDA for schistosomiasis and STH history varied between the districts surveyed, and also between islands within a district. Of the nine districts surveyed, existing and trained CMDs were found in Bugiri, Jinja, Mayuge, Mukono and Kalangala, whereas few were available in Masaka and Wakiso; data on CMDs were not available for Mpigi and Rakai. Treatment was only regularly administered to the community at large in Jinja; the last MDA had been in 2009 for Mukono, 2008 for Kalangala, Mayuge and Masaka and 2007 for Bugiri and Wakiso. As with the CMD survey, information on treatment delivery was not available for Mpigi and Rakai. Overall, the proportion of children who reported to have received treatment in the past year was 49.7% (number treated [n] = 1122; number surveyed [*N*] = 2256; 95% confidence intervals [CI] = 47.7-51.8%). Of these, 60.2% reported having received the treatment in a school-based setting (n = 676; *N = *1122; 95% CI = 57.3-63.1%).

In terms of capacity building and on-going treatment delivery, CMDs were trained on each of the islands visited. In mainland areas, CMDs were trained in individual landing sites, to overcome logistical difficulties associated with transport between sites not linked by paved roads. The exception to this was in Masaka, where a dearth of volunteers resulted in some villages remaining without CMDs. During this survey, all villages surveyed were provided with medication for schistosomiasis and STH, and participants in the parasitological portion of the survey were treated for these diseases. Overall, of a total population of 193,875 people, 157,814 were estimated to be older than 5 years, and thus were targeted for treatment. Of these, 108,305 people were successfully treated, during and immediately after the survey achieving treatment coverage of 68.6%. In total, 192,820 tablets of ALB were distributed to the CMDs, along with 426,098 tablets of PZQ.

### Parasitological surveys

Overall, 5016 school-age children were asked to participate in the parasitological surveys, of which 2578 were boys, 2428 were girls and 10 did not have their sex recorded, the mean age was 12.5 years. A total of 4540 children provided a stool sample, of which 4534 were examined for presence of *S. mansoni *eggs and all were read for STH prevalence. As for malaria diagnosis, a total of 3712 children provided a fingerprick blood sample.

Overall prevalence of *S. mansoni *was found to be 40.8% (n = 1850; *N = *4534; 95% CI = 39.4- 42.3%). Prevalence of malaria was higher, at 46.4% (n = 1724; *N = *3712; 95% CI = 44.8-48.1%). Moreover, co-infection of schistosomiasis and malaria was relatively common, reaching 23.5% prevalence (n = 839; *N = *3569; 95% CI = 22.1-24.9%). Prevalence of STH was 14.9% for hookworm (n = 674; *N = *4540; 95% CI = 13.8-15.9%), 4.3% for *Ascaris lumbricoides *(n = 196; *N = *4540; 95% CI = 3.7-5.0%) and 11.9% for *Trichuris trichiura *(n = 538; *N = *4540; 95% CI = 10.9-12.8%). The breakdown of prevalence by district for schistosomiasis, malaria and co-infection can be found in Table 2, and in Table 3 for STH prevalence.

**Table 2:** Summary of the prevalence of *S. mansoni*, malaria and co-infections by district and overall

	INFECTION								
DISTRICT	*S. mansoni*			*P. falciparum*			Co-infection		
	
	n	N	Prevalence (95% CIs)	n	N	Prevalence (95% CIs)	n	N	Prevalence (95% CIs)
**Bugiri**	363	484	75.00 (70.89-78.80)	282	495	57.00 (52.48-61.38)	191	440	43.41 (38.72-48.19)

**Jinja**	114	170	67.06 (59.45-74.06)	93	161	57.76 (49.74-65.50)	63	156	40.38 (32.61-48.53)

**Kalangala**	302	962	31.39 (28.47-34.43)	307	777	39.51 (36.05-43.05)	119	814	14.62 (12.26-17.24)

**Masaka**	41	218	18.81 (13.85-24.64)	125	220	56.82 (50.00-63.46)	25	210	11.90 (7.85-17.07)

**Mayuge**	241	356	67.70 (62.57-72.53)	144	358	40.22 (35.10-45.51)	90	340	26.47 (21.86-31.50)

**Mpigi**	64	212	30.19 (24.09-36.85)	104	211	49.29 (42.36-56.24)	34	200	17.00 (12.07-22.94)

**Mukono**	586	1134	51.68 (48.72-54.62)	586	1165	50.30 (47.39-53.21)	285	1099	25.93 (23.36-28.63)

**Rakai**	5	667	0.75 (0.24-1.74)	NA	NA	NA	NA	NA	NA

**Wakiso**	134	331	40.48 (35.15-45.99)	83	325	25.54 (20.89-30.64)	32	310	10.32 (7.17-14.26)

**TOTAL**	**1850**	**4534**	**40.80** **(39.37-42.25)**	**1724**	**3712**	**46.44** **(44.83-48.06)**	**839**	**3569**	**23.51** **(22.12-24.93)**

"n" refers to the number of positive cases, "N" is the total number of children surveyed. "CIs" stand for confidence intervals.

**Table 3:** Prevalence of STH infection, per district and overall

	STH INFECTION								
DISTRICT		Hookworm		*Ascaris lumbricoides*		*Trichuris trichiura*		STH overall	
		
	N	n	Prevalence (95% CIs)	n	Prevalence (95% CIs)	n	Prevalence (95% CIs)	n	Prevalence (95% CIs)
**Bugiri**	484	73	15.08 (12.01-18.59)	23	4.75 (3.04-7.05)	112	23.14 (19.45-27.16)	184	38.02 (33.67-42.51)

**Jinja**	173	11	6.36 (3.22-11.09)	0	0.00 (0.00-2.11)	2	1.16 (0.14-4.11)	13	7.51 (4.06-12.51)

**Kalangala**	962	120	12.47 (10.45-14.73)	54	5.61 (4.24-7.26)	98	10.19 (8.35-12.27)	207	21.52 (18.96-24.25)

**Masaka**	218	28	12.84 (8.71-18.03)	30	13.76 (9.48-19.06)	96	44.04 (37.34-50.90)	120	55.05 (48.18-61.77)

**Mayuge**	356	84	23.60 (19.28-28.36)	16	4.49 (2.59-7.20)	17	4.78 (2.81-7.54)	100	28.09 (23.48-33.07)

**Mpigi**	212	42	19.81 (14.67-25.82)	6	2.83 (1.05-6.06)	32	15.09 (10.56-20.64)	68	32.08 (25.85-38.81)

**Mukono**	1137	162	14.25 (12.27-16.42)	36	3.17 (2.23-4.36)	87	7.65 (6.17-9.35)	251	22.08 (19.70-24.60)

**Rakai**	667	78	11.69 (9.35-14.38)	21	3.15 (1.96-4.77)	68	10.19 (8.00-12.75)	138	20.69 (17.68-23.97)

**Wakiso**	331	76	22.96 (18.54-27.87)	10	3.02 (1.46-5.49)	26	7.85 (5.20-11.30)	101	30.51 (25.60-35.78)

**TOTAL**	**4540**	**674**	**14.85** **(13.82-15.91)**	**196**	**4.32** **(3.74-4.95)**	**538**	**11.85** **(10.92-12.83)**	**1182**	**26.04** **(24.76-27.34)**

"N" is the total number of children surveyed for STH, "n" refers to the number of positive cases and "CIs" stand for confidence intervals.

The prevalence of co-infections of schistosomiasis and STH and of malaria and STH was also calculated by district (Tables 4 and 5 respectively). Overall prevalence of co-infection with schistosomiasis and STH was 13.3% (n = 603; *N = *4534; 95% CI = 12.3-14.3%), although Bugiri District had co-infection levels as high as 33.1% (n = 160; N = 484; 95% CI = 28.9-37.5%). Overall prevalence of co-infection with malaria and STH was 13.8% (n = 493; *N = *3574; 95% CI = 12.7-15.0%).

**Table 4:** Prevalence of STH and schistosomiasis co-infection, per district and overall

	STH CO-INFECTION WITH SCHISTOSOMIASIS								
DISTRICT		Hookworm		*Ascaris lumbricoides*		*Trichuris trichiura*		STH overall	
		
	N	n	Prevalence (95% CIs)	n	Prevalence (95% CIs)	n	Prevalence (95% CIs)	n	Prevalence (95% CIs)
**Bugiri**	484	61	12.60 (9.78-15.89)	20	4.13 (2.54-6.31)	101	20.87 (17.33-24.76)	160	33.06 (28.88-37.45)

**Jinja**	170	7	4.12 (1.67-8.30)	0	0.00 (0.00-2.15)	2	1.18 (0.14-4.19)	9	5.29 (2.45-9.81)

**Kalangala**	962	63	6.55 (5.07-8.30)	23	2.39 (1.52-3.57)	45	4.68 (3.43-6.21)	90	9.36 (7.59-11.37)

**Masaka**	218	4	1.83 (0.50-4.63)	7	3.21 (1.30-6.50)	22	10.09 (6.43-14.88)	26	11.93 (7.94-16.99)

**Mayuge**	356	66	18.54 (14.64-22.97)	12	3.37 (1.75-5.81)	13	3.65 (1.96-6.16)	78	21.91 (17.72-26.57)

**Mpigi**	212	12	5.66 (2.96-9.68)	3	1.42 (0.29-4.08)	20	9.43 (5.86-14.19)	29	13.68 (9.36-19.05)

**Mukono**	1134	100	8.82 (7.23-10.62)	25	2.20 (1.43-3.24)	60	5.29 (4.06-6.76)	162	14.29 (12.30-16.46)

**Rakai**	667	2	0.30 (0.04-1.08)	0	0.00 (0.00-0.55)	0	0.00 (0.00-0.55)	2	0.30 (0.04-1.08)

**Wakiso**	331	37	11.18 (7.99-15.08)	5	1.51 (0.49-3.49	9	2.72 (1.25-5.10)	47	14.20 (10.62-18.43)

**TOTAL**	**4534**	**352**	**7.76** **(7.00-8.58)**	**95**	**2.10** **(1.70-2.56)**	**272**	**6.00** **(5.32-6.73)**	**603**	**13.30** **(12.32-14.32)**

"N" is the total number of children surveyed for both schistosomiasis and STH infection, "n" refers to the number of co-infected cases. "CIs" stand for confidence intervals.

**Table 5:** Prevalence of STH and malaria co-infection, per district and overall

	STH CO-INFECTION WITH MALARIA								
DISTRICT		Hookworm		*Ascaris lumbricoides*		*Trichuris trichiura*		STH overall	
		
	N	n	Prevalence (95% CIs)	n	Prevalence (95% CIs)	n	Prevalence (95% CIs)	n	Prevalence (95% CIs)
**Bugiri**	440	42	9.55 (6.97-12.68)	10	2.27 (1.10-4.14)	65	14.78 (11.59-18.44)	102	23.18 (19.32-27.41)

**Jinja**	158	8	5.06 (2.21-9.73)	0	0.00 (0.00-2.31)	0	0.00 (0.00-2.31)	8	5.06 (2.21-9.73)

**Kalangala**	814	50	6.14 (4.59-8.02)	22	2.70 (1.70-4.06)	36	4.42 (3.12-6.07)	82	10.07 (8.09-12.35)

**Masaka**	210	23	10.95 (7.07-15.98)	19	9.05 (5.54-13.77)	56	26.67 (20.82-33.19)	73	34.76 (28.34-41.62)

**Mayuge**	340	36	10.59 (7.53-14.36)	6	1.76 (0.65-3.80)	3	0.88 (0.18-2.56)	42	12.35 (9.05-16.33)

**Mpigi**	200	23	11.50 (7.43-16.75)	4	2.00 (0.55-5.04)	14	7.00 (3.88-11.47)	35	17.50 (12.50-23.49)

**Mukono**	1102	90	8.17 (6.62-9.94)	23	2.09 (1.33-3.12)	35	3.18 (2.22-4.39)	126	11.43 (9.61-13.46)

**Wakiso**	310	17	5.48 (3.23-8.64)	2	0.65 (0.08-2.31)	7	2.26 (0.91-4.60)	25	8.06 (5.29-11.67)

**TOTAL**	**3574**	**289**	**8.09** **(7.21-9.03)**	**86**	**2.41** **(1.93-2.96)**	**216**	**6.04** **(5.28-6.88)**	**493**	**13.79** **(12.68-14.97)**

"N" is the total number of children surveyed for both malaria and STH infection, "n" refers to the number of co-infected cases. "CIs" stand for confidence intervals.

### Mapping disease prevalence

**Figure 2:**
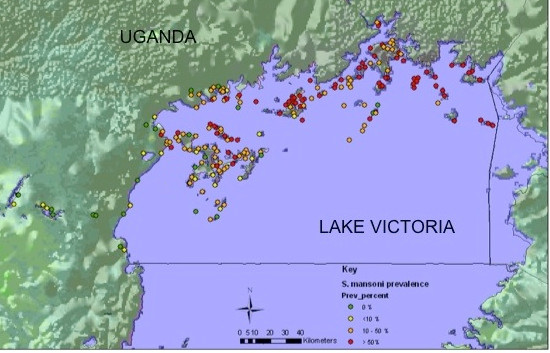
**Distribution and prevalence of *Schistosoma mansoni *infection**. Distribution and prevalence of *Schistosoma mansoni *infection in school-age children across the surveyed districts.

**Figure 3:**
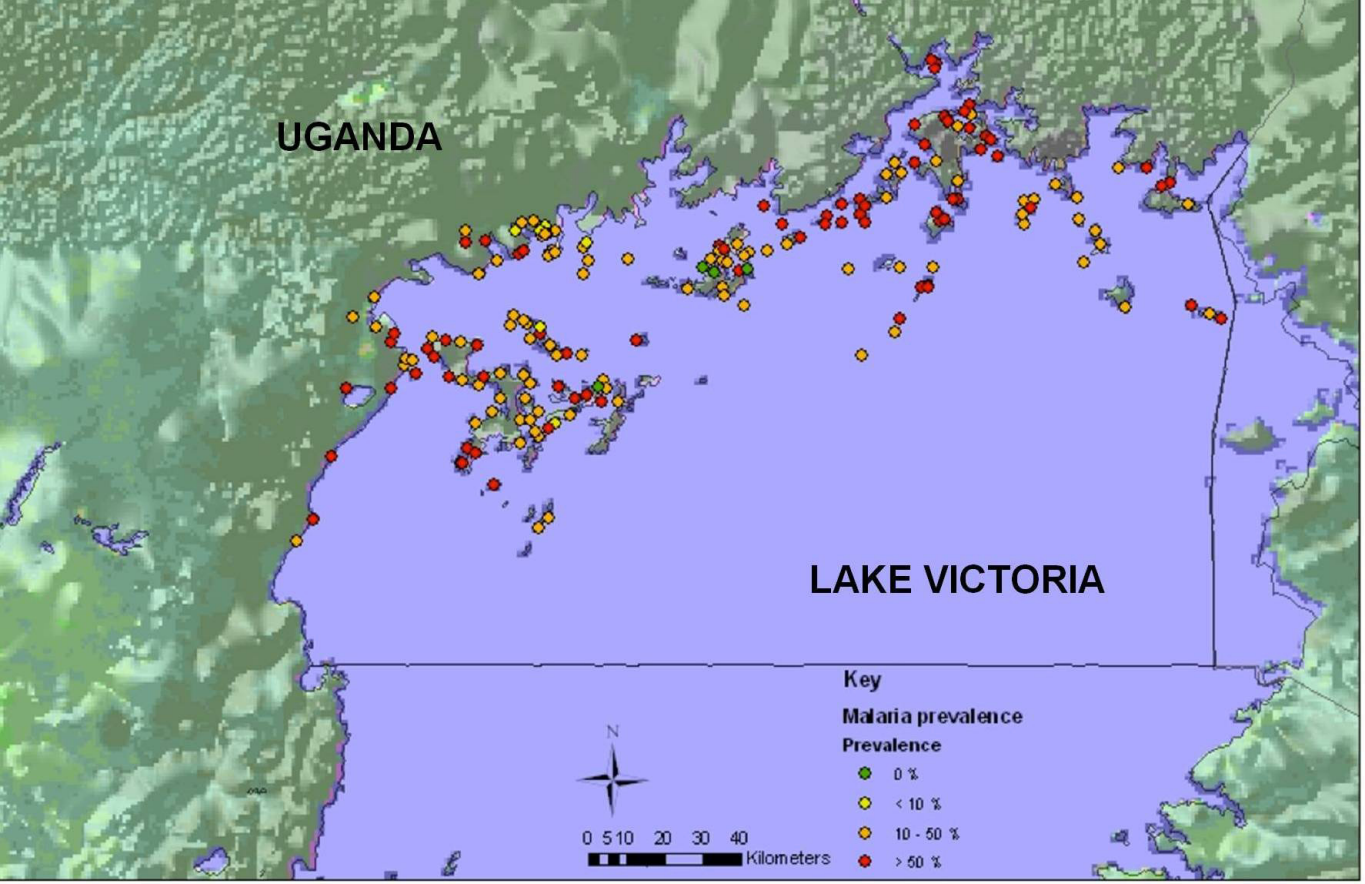
**Distribution and prevalence of *Plasmodium falciparum *infection**. Distribution and prevalence of *Plasmodium falciparum *infection in school-age children across the surveyed districts.

The distribution of both schistosomiasis and malaria was heterogeneous across the region (please refer to Figure 2 for the map of intestinal schistosomiasis and Figure 3 for the map of malaria prevalence levels).

**Figure 4:**
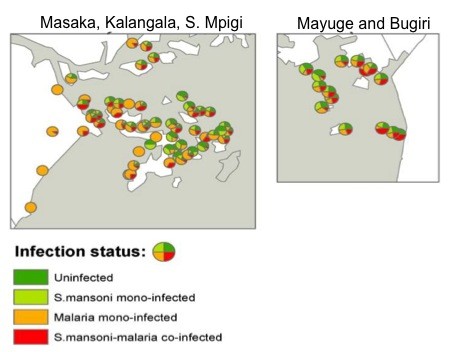
**Distribution and prevalence of *Schistosoma mansoni *and *Plasmodium falciparum *co-infection**. Distribution and prevalence of *Schistosoma mansoni *and *Plasmodium falciparum *co-infection across western districts (Kalangala, Masaka and southern Mpigi; left-hand image) and eastern districts (Mayuge and Bugiri; right-hand image).

There was also variation in the distribution of co-infections, which were mapped in detail only for the extreme Western-most (Kalangala, Masaka and part of Mpigi) and Eastern-most (Mayuge and Bugiri) districts. Figure 4 shows the distribution and proportion of uninfected children, mono-infected children (with either *S. mansoni *or *Plasmodium *spp.) and co-infected cases.

### Statistical analysis of questionnaire data and infection predictors

The multivariate logistical stepwise regression modeling of risk factors for intestinal schistosomiasis and malaria revealed a clear association with age in both cases, with infection risk increasing with age for intestinal schistosomiasis (odds ratio [OR] = 1.03; 95% CI = 1.00-1.06; *P *= 0.024) and decreasing with age for malaria (OR = 0.97; 95% CI = 0.95-0.99; *P = *0.005). Both *S. mansoni *infection and *Plasmodium spp*. infection were also associated with increased likelihood of co-infection with hookworm (OR = 1.50; 95% CI = 1.12-2.02; *P = *0.008 and OR = 1.59; 95% CI = 1.22-2.07; *P <*0.001, respectively). Intestinal schistosomiasis was also associated with increased likelihood of infection with *T. trichiura *(OR = 1.39; 95% CI = 1.05-1.85; *P = *0.015), but strongly negatively associated with the presence of a household latrine (OR = 0.50; 95% CI = 0.39-0.64; *P <*0.001). For malaria, symptoms of fever on the day of the surveys were a predictor for a positive malaria diagnosis (OR = 1.55; 95% CI = 1.17-2.04; *P = *0.002). All the variables that were statistically significant or approaching significance are presented in Table 6.

**Table 6:** Multivariate model selected by stepwise logistic regression results for infection with either *S. mansoni *or malaria

Response variable	Explanatory variable (baseline; category)	Odds ratio (95% CIs)	p-value
*S. mansoni*	Sex (male; female)	0.81 (0.66-0.99)	0.039
	
	Age (continuous; +1 year)	1.03 (1.00-1.06)	0.032
	
	Hookworm infection (uninfected; infected)	1.50 (1.12-2.02)	0.007
	
	*Trichuris *infection (infected; uninfected)	1.39 (1.05-1.85)	0.022
	
	Household latrine present? (no; yes)	0.50 (0.39-0.64)	< 0.001
	
	Enrolled in school? (no; yes)	2.93 (2.11-4.06)	< 0.001
	
	Education level of household head (plus one category*)	1.10 (1.04-1.17)	0.003
	
	Source of household water (clean; river or lake)	1.28 (0.99-1.66)	0.062

Malaria	Sex (male; female)	0.95 (0.71-1.15)	0.61
	
	Age (continuous; +1)	0.97 (0.95-0.99)	0.005
	
	Hookworm infection (uninfected; infected)	1.59 (1.22-2.07)	< 0.001
	
	Are you feverish today? (no; yes)	1.55 (1.17-2.04)	0.002

Includes variables selected for inclusion in the model according to AIC stepwise regression (N = 1784 and 1802 children for schistosomiasis and malaria respectively), and controlling for random effects at the village level. *For details, please see the questionnaire, which is available as Additional File 1.

Factors such as bednet usage, socio-economic status (SES) and absenteeism were examined more closely through direct multi- and univariate logistical regression analyses. Controlling for age and gender, there was no significant association between reported bednet use and infection status with malaria (*N = *2052). At a univariate level, SES was not found to be significantly associated with malaria, either as an overall metric or within the individual measured categories (presence of electricity, solar power, latrine, landline or mobile phone in the household). However, infection with schistosomiasis was strongly predicted by low SES, whereby the addition of an item in the household resulted in drop of odds of being found infected (OR = 0.74; 95% CI = *P *= < 0.001), and specifically by lack of a household latrine, whereby children residing in households with latrines were less likely to be infected (OR = 0.47; 95% CI = 0.38-0.59; *P = *< 0.001), as for the stepwise analysis. Schistosomiasis, malaria and/or STH ("being ill") prevalence was found to be negatively associated with presence of a household latrine (OR = 0.65; 95% CI = 0.49-0.85; *P = *0.002), with household ownership of a mobile phone (OR = 0.71; 95% CI = 0.55-0.91; *P = *0.007) and with an increased overall SES metric (OR = 0.68; 95% CI = 0.58-0.81; *P = *< 0.001). Sample size was 1898 children for all SES analyses. Children who were positive for schistosomiasis, or ill overall, were more likely to be in school (OR = 2.75; 95% CI = 2.02-3.74; *P = *< 0.001, and OR = 1.83; 95% CI = 1.31-2.55; *P *= < 0.001, respectively), meaning that children who reported going to school regularly were at higher risk of disease, controlling for age and gender. On the other hand, after analysis of responses in the questionnaire (*N *= 840 complete questionnaires), children report malaria to be the primary reason for absenteeism (39.6%; 95% CI = 36.3-43.0%), although absenteeism was not a statistically significant predictor of malaria infection during regression analysis.

## Discussion

### Treatment availability and capacity building

Uganda's National Control Programme for combating intestinal schistosomiasis and STHs has been active since 2003, and although regular monitoring has occurred at intervals since then, this is the first comprehensive overview of the status of treatment infrastructure and health capacity on the districts adjacent to Lake Victoria. The results here paint a mixed picture; on the one hand, some villages appear to function well in terms of drug availability, regular training of CMDs and administration of mass treatment. However, there were also locations, such as much of Wakiso district islands, where people were sceptical about the efficacy or need to take PZQ, while in Masaka district there was a noticeable absence of volunteers willing to take on the role of CMD. The reasons given for refusal included the distance between sites and the lack of adequate funding for travel. Even within a district with generally good treatment coverage, there were islands that did not conform to what was being observed elsewhere in the district. For example, on one island in Kalangala district, all inhabitants refused to take PZQ, citing experience of severe side-effects after swallowing the drugs during an earlier treatment campaign. High perceived risk of treatment has been associated with non-compliance with treatment in other studies [20], and so may limit the effectiveness of future treatment campaigns where side-effects have been noted. These heterogeneities in responses to treatment, added to the epidemiological variations seen across the region in terms of parasite prevalence, indicate the challenges to maintaining effective and efficient disease control. One limitation of this study was the lack of funding for follow-up visits to all of the communities surveyed in order to check up on the actions of the newly-trained CMDs and to see whether MDA was now being carried out more regularly.

### High disease prevalence and observation of co-infection

The eastern part of Lake Victoria is known to be a highly endemic zone for intestinal schistosomiasis [5,21]. In 2007, Masaka and Mpigi districts were added to the roster of districts receiving MDA, based on data showing increased disease levels as compared to the original 2003 baseline data [22]. This current survey revealed that despite regular chemotherapy and educational interventions, levels of intestinal schistosomiasis are still very high. The only exception is Rakai district, which continues to have low, almost negligible, levels of *S. mansoni*. The data here also support earlier evidence of a geographical west to east cline along Uganda's Lake Victoria shoreline of increasing schistosomiasis prevalence, although the observation of significant local heterogeneities should be taken into consideration. The reasons for this purported cline are not fully understood, but may relate to climatic variation along the lakeshore, or indeed differences in population density or sanitation infrastructure. Large-scale variation in prevalence may be more a residue of sampling effort or treatment history, and indeed if given too much weight, may confound smaller-scale patterns of variation which are crucial for local disease management recommendations [23,24].

The patterns of distributions of STH and malaria infections are less geographically skewed and more evenly distributed by district, but again, there are exceptions. Masaka district has significantly higher prevalence of *T. trichiura *infection than the overall average, for example. This is somewhat surprising, since all three STHs are successfully treated with albendazole, and prevalence of hookworm and *A. lumbricoides *are not noticeably elevated. One possibility is the observation of particularly poor sanitary and hygiene infrastructure in many locations along Masaka's Lake Victoria shoreline, encouraging STH transmission; if soil conditions here are more suitable for persistence of *T. trichiura *eggs than in other areas, this could go some way to explaining why the infection rate of this species of helminth is higher in Masaka than elsewhere. Indeed, a soil map of Uganda shows mineral hydropmorphic soils in Masaka district, and extending to Rakai, in contrast to ferrallitic soils along the rest of the Lake Victoria shoreline in Uganda [25].

In terms of malaria prevalence, Wakiso and Kalangala districts have significantly lower levels of *Plasmodium *spp. than the overall average. For the former, this may be due to the generally more urban environment of the district, as well as its proximity to cities such as Entebbe and Kampala, where health infrastructure is better. Kalangala is a rural and remote district with many islands and limited connectivity to health centres; therefore the lower malaria prevalence is likely due to environmental factors, such as unsuitable habitat for the mosquito vectors. Indeed, some environmental modeling work has shown that like intestinal schistosomiasis, malaria risk is predicted to be slightly lower in the western portions of Lake Victoria as opposed to the eastern [26,27]. However, future surveys should also focus on training and education for behavioural control mechanisms such as bed net possession and insecticide use, neither of which was directly measured in this survey, to see whether these factors are contributing to the lower malarial prevalence observed in Kalangala.

The relatively high incidence of malaria and schistosomiasis co-infections, particularly in districts such as Bugiri and Jinja, was a very significant finding. Bugiri District also had high levels of co-infection of STH with both schistosomiasis and malaria, marking it out as a key region for future surveys of co-infection incidence and also the health effects of infection with multiple parasites. There is a large body of research on the immune activity of patients co-infected with different parasites, with some evidence suggesting malaria leads to increased susceptibility to *S. mansoni *infection [28,29], but also reports of modulation of malaria via up-regulation of the immune system as a result of helminth infection [30]. In these cases, treating for the helminth can result in a significant increase in the burden of malaria, further complicating decisions regarding appropriate control measures. On the other hand, certain areas within individual districts had notably high levels of one infection as compared to another, such as high malaria but low schistosomiasis prevalence in some of the islands in Mayuge district, as compared to low malaria and high schistosomiasis prevalence on the Koome islands in Mukono district. Managing these complex interactions is one of the many challenges facing existing and future control measures for NTDs in the Lake Victoria region.

### Challenges for control of NTDs in the context of remote island communities

Several points can be made relating to the current status of disease distribution and control interventions based on the results of these surveys. Firstly, it is clear that prevalence of intestinal schistosomiasis, STH and malaria continue to be high in many of the islands and the districts bordering on Lake Victoria. Consequently, the National Control Programme, which has been successful in reducing intensity and disease-related morbidity [22], needs to improve treatment coverage and efficacy of existing control measures in the areas studied.

In the context of the islands of Lake Victoria, logistical challenges were identified as a huge obstacle for regular training of CMDs, and timely and frequent delivery of drugs. Community health workers are often compelled to travel, by wooden canoes for several hours, to reach the outer islands in the lake; fuel for canoe engines is expensive, and often unavailable. Another challenge is the high level of population itinerancy and migration throughout the region; this has been observed in other studies [21], and migration was cited as a key reason for treatment coverage during the survey only being estimated at approximately 70%, although residency length was not included in the questionnaire presented here. This highlights the need to include measures of itinerancy in future surveys, for accurate identification of the scale of population movement in the region. A solution would be to provide adequate funding to local health officers for local travel; however, even if such funds were available, supervision and accountability of the system must simultaneously improve in order to ensure the investment reaches the people who need it the most.

The question of funding, and in particular, sustainability of control initiatives, is intrinsic to creating an effective yet efficient programme for reducing disease burden. Although chemotherapy has been the mainstay of control tactics in the last decade, it is clear that MDA alone will not sufficiently eliminate transmission of schistosomiasis, especially in hyper-endemic areas such as the Lake Victoria islands. What is required is a combination of political will, education and improvements in access to sanitation and clean water, alongside a committed and consistent chemotherapy regime [31]. Indeed, such an integrated approach has proved successful for the on-going and sustainable control of helminthes and intestinal parasites in other island settings, such as the Seychelles [32], where improved socio-economic status has also contributed to reduction in disease. Given the correlation of such factors with increased disease risk in this study, overall economic development may also prove to be highly beneficial in this setting. However, in Lake Victoria, the scale of the problem is larger; moreover, given the levels of co-infection with malaria observed here and in other studies [33], any control strategy in East Africa will also have to take into account the possibility, and indeed relatively high probability, of co-infection with malaria.

As with intestinal helminthes, controlling malaria may also be achievable through a combination of access to treatment, distribution of bednets and indoor residual spraying. We mobilized communities to select CMDs who were immediately trained and started distributing medicines and disseminating health education messages. These CMDs could also be trained to deliver malaria control interventions such as distributing insecticide-treated mosquito nets and ACTs among these hard-to-reach communities, therefore providing additional health benefits without the need to identify and/or train additional health workers. Bednets may also have additional protective benefits against other infections, such as lymphatic filariasis, also transmitted via mosquito vectors [34]. Thus integrated monitoring of schistosomiasis, STH and malaria is indeed as appealing as the widely advocated integrated delivery of preventive chemotherapy (PCT) packages against NTDs [2,35]. With the ever-limited resources, especially in sub-Saharan countries, it is clear that integrating surveys and control of intestinal helminthes along with malaria is likely to provide the most efficient method of scaling up the control of the targeted multiple parasitic infections across a regional scale.

## Competing interests

The authors declare that they have no competing interests.

## Authors' contributions

NBK conceived the study, coordinated the field research, assisted in the analysis and oversaw the writing of the manuscript. CJS participated in the field surveys, conducted the geospatial analyses and wrote the first draft of the manuscript. JCS-F conducted the statistical analyses and the writing of the manuscript. FMF was involved in the design of the study, the planning of the fieldwork and edited the manuscript. JRS participated in the field surveys, assisted in the analyses and in the writing of the first draft of the manuscript. AT assisted in the study design and implementation and edited the manuscript. AF was involved in the study conception, design and implementation, and edited the manuscript. All authors read and approved the final manuscript.

## Supplementary Material

Additional file 1 **Sample questionnaire sheet**. The questionnaire sheet was used to obtain information on demographics, socio-economic status, health behaviour and absenteeism. Click here for file
