# Supplementary material for: Integrated prevalence mapping of schistosomiasis, soil-transmitted helminthiasis and malaria in lakeside and island communities in Lake Victoria, Uganda
Source: Parasit Vectors. 2011 Dec 13;4:232. doi: 10.1186/1756-3305-4-232 (PMC3270004; doi:10.1186/1756-3305-4-232)
Supplement: Additional file 1 — Sample questionnaire sheet. The questionnaire sheet was used to obtain information on demographics, socio-economic status, health behaviour and absenteeism. [file 1756-3305-4-232-S1.PDF]

UGANDA SCHOOL MALARIA AND SCHISTOSOMIASIS SURVEY, 2009

GPS Cordinates:
N:..E:..Elev:.................................

CHILD INFORMATION	
**Village code:** |____|____|____|	**Village name:**
**District Code:** [___]___]___]	**District name:**
**Child ID** |___|___|___|___|___|___|___|	**Date of visit:** |___|___|/|___|___|/|___|___| ***day month year***
**Child's last name**	**Child's first name**
**Child's initials**	**Date of birth** |___|___|/|___|___|/|___|___| ***day month year*** 99/99/99=Not known
**Age:** ||____| **years**	**Gender:** ☐ **Male** ☐ **Female**
**Parent/guardian's last name**	**Parent/guardian's first name**

HEALTH INFORMATION	
**Haemoglobin:** |___|___|___| g/L	**Auxiliary temperature:** |___|___|___| . |___| oC
**Malaria RDT used:**	**Malaria RDT:** Positive ☐ Negative ☐ Unreadable ☐
**Blood slide taken:** ☐ Yes ☐ No	**Stool slide taken:** ☐ Yes ☐ No

RESIDENCE

A1. What is the name of the village you reside in now? / ______________________________

A2. Have you ever been enrolled in school? 1= Yes 0= No

A 3. Which school do you go to? ____

HOUSEHOLD WEALTH INFORMATION

C1. What is the highest level of education attained by the household head? **Read out options, only enter one answer**
.... 1 = No education; 2 = Primary incomplete; 3 = Primary complete; 4 = Secondary incomplete; 5 = Secondary complete or above; 6 = Don't know

C2. What type of wall does your house have? **Read out options, only enter one answer**
.... 1=Stone or bricks or cement; 2=Clay or mud; 3=Wood; 4=Iron sheets; 5=Other *specify* [____________________]

C3. What type of flooring is there in the household head's house? **Read out options, only enter one answer**
.... [__] 1=Cement, tiles or linoleum; 2=Wooden planks; 3=Earth or sand; 4=Iron sheets; 5=Other *specify*[____________________]

C4. What is the main source of water for drinking or cooking in this household? **Read out options, only enter one answer**
.... [__] 1=Piped/tap water; 2=Borehole or well; 3=Rain water; 4=Stream or river; 5=Bought; 6=Bottled water; 7=Others i.e LAKE (all 7s are lake)
.... *specify* [________________________]

C5. In your house, are there any of the following? **Read out and fill with 1= Yes; 0 = N:**
Electricity .... [__]
Solar power .... [__]
Flush toilet .... [__]

Pit latrine .... [__]
No toilet or latrine/bush/field.. [__]
Telephone; landline .... [__]
Telephone; mobile phone .... [__]

BEDNET USE

D1. Do you normally sleep under a bednet? **Read out options, only enter one answer**
.... [__] 1 = Yes; 0= No; 2= Don't know

D2. Did you sleep under a bed net last night? **Read out options, only enter one answer**
.... [__] 1 = Yes; 0 = No; 2= Don't know

D3. What is the colour of your bed net? **Read out options, only enter one answer**
.... [__] 1 = Blue; 2 = Green; 3 = White; 4 = Red; 5 = Others.. *specify* [____________________] 6= N/A

D4. When did you receive your bed net? **Read out options, only enter one answer**
.... [__] 1 = This school term; 2 = Last school term; 3 = Last school year; 4=Before last school year 5 = N/A

D5. From where did you receive your bednet? **Read out options, only enter one answer**
.... [__] 1 = School; 2 = Health center; 3 = Home; 4 = Community programme; 5 = Others *specify* [] 6= N/A

D6. Has the net ever been treated with an insecticide? /**Read out options, only enter one answer**
.... [__] 1 = Yes; 2 = No; 3 = Don't know 4= N/A

D7. If yes in D6, where was the net treated? **Read out options, only enter one answer**
.... [__] 1 = Pretreated net; 2 = Treated at home; 3 = Don't know; 4 = Other Specify / [____________________] 5= N/A

DEWORMING USE

E1. Have you received treatment for worms in the last year? **Read out options, only enter one answer**
.... [__] 1 = Yes; 0= No; 2= Don't know

E2. If yes, where did you receive treatment? **Read out options, only enter one answer**
.... [__] 1 = School; 2 = Health center; 3 = Home; 4 = Community programme; 5 = Others *specify* []

ABSENTEEISM and RECENT ILLNESS

F1. During this school term, have you been absent from school due to illness? **Read out options, only enter one answer**
.... [__] 1 = Yes; 0= No; 2= Don't know

F2. In the last 2 weeks, have you been absent from school due to illness? **Read out options, only enter one answer**
.... [__] 1 = Yes; 0 = No; 2= Don't know

F3. If absent in the last 2 weeks due to illness, what was illness ? **Read out options, only enter one answer**
.... [__] 1 = Headache / 2=Stomach ache/ 3=Malaria/ 4=Vomiting/ 5=Diarrhoea/ 6=Cough; 7 = Others *specify* [____________________]

F4. Do you have fever or hot body today? **Read out options, only enter one answer**
.... [__] 1 = Yes; 0= No; 2= Don't know

F5. In the last 2 weeks, have you had fever or hot body? **Read out options, only enter one answer**
.... [__] 1 = Yes; 0 = No; 2= Don't know
